# Bananas, coffee and palm oil: The trade of agricultural commodities in the framework of the EU-Colombia free trade agreement

**DOI:** 10.1371/journal.pone.0256242

**Published:** 2021-08-24

**Authors:** Julieth P. Cubillos T., Béla Soltész, László Vasa

**Affiliations:** 1 Hungarian University of Agriculture and Life Science, Gödöllő, Hungary; 2 Hungary and Institute for Foreign Affairs and Trade, Eötvös Loránt University, Budapest, Hungary; 3 Hungary and Institute for Foreign Affairs and Trade, Széchenyi István University, Győr, Hungary; Wroclaw University of Economics and Business, POLAND

## Abstract

Generally, research and studies about commodities focus on price trends, analysis in terms of international competitiveness, market position structure, rate of net exports, market share, and concentration index. This paper has developed an analysis of the most influential agricultural commodities traded from Colombia to European Union, which are bananas, coffee, and palm oil. Analyzing the economic and commercial effects in two traditional agricultural commodities from Colombia (bananas and coffee) with the rise of palm oil as a commodity in the trade relation with its partner; the European Union. The structure draws from the overview of general aspects and the behavior of Colombian foreign trade, as diversification of export products and trade partners, to focus on the characteristics of the trade relationship between the European Union and Colombia. The aim is analyze the proportional relation between bananas, coffee, and palm oil exported to the EU, according to three indicators, the volume of production, exports share, and trade value, from 2008 until 2019, identifying the trends before and after the implementation of the free trade agreement. Finally, with the coefficient correlation, determine the agricultural commodity that has the strongest and positive relationship with the total agricultural exports value from Colombia to the European Union.

## Introduction

All along its history, Colombia has been specialized in the primary sector in international trade, based on primary goods, exports of natural resources, and raw materials without value added. Once predominantly a coffee-based economy, between 1930 and 1989, the coffee share was around 60.2% in total national exports, and other agricultural commodities (bananas, flowers, meat, sugar, and tobacco) were 5.9%, the mining sector and oil had a share of 11.5%, and the manufacturing sector was 22.3%, according to [1].

With the economic aperture, Colombian export performance began to change in the composition of the commodities traded. By 2000, coffee accounted for only 8.43% of foreign exchange earnings, while oil and mining products jumped to 35.34%. By 2018, the reduction of agricultural percentage in national exports was notorious, with the participation of 12%, and a coffee export share of 3.92%.

It could be considered that this situation was because other products, from the industrial sector with value added, began to be traded, but this was not the situation. After the aperture, traditional exports represented half, and even slightly more, of total exports, which suggests that the economy is anchored in these traditional products. Nevertheless, the dependency “began to concentrate on the exploitation of natural and mining resources” [2], and the agriculture sector was lagging, which is acknowledged in the export trade from Colombia to European Union (EU) as well.

It is necessary to analyze the performance of the Colombian agricultural sector in the export trade because of its historical and social importance in the national economy, and the contribution of this sector to the development of rural areas. Moreover, despite the reduction in share of the agricultural sector in national exports, Colombian agricultural commodities continue with a leading role in international trades, since the country is among one of the main worldwide exporters of five agricultural commodities (flowers, coffee, bananas, palm oil, and sugar cane).

In the European Union, bananas, coffee, and palm oil are the most imported agricultural commodities from Colombia; 80% of the total production of Colombian bananas go to the EU market, and palm oil export value to the EU “increased more than 252% from 2012 to 2018” [3]. Nowadays, with the free trade agreement, the European Union has identified potential sectors to invest in in Colombia; oriented direct investment in tourism, infrastructure, and agro-industry, bestowing an important role to palm oil production.

Considering this context, the hypothesis was posited about ‘the rise of the palm oil export trade, from Colombia to European Union, has reduced the participation and export value of the Colombian traditional agricultural commodities (Bananas and Coffee)’. This hypothesis is evaluated in this research paper.

## Literature review and context

Recent research on the trade relationship between Colombia and European Union have been a focus on the impact of the free trade agreement between these two partners, evaluating the changes in specific economic sectors like tourism, industry, and agriculture, and, moreover, foreign policy agendas, and cooperation and investment trends. Reports from public institutions such as the European Parliamentary Research Service (EPRS), national statistics departments, ministries of trade, and organizations such as ElanBiz—business and investment opportunities in Latin America—generated relevant information regarding the current trade relationship.

There are global studies on the impact of preferential trade agreements, as [4] who did an analysis of 189 countries to identify the global production network and product-base coverage index, calculated in terms of intermediate good, final goods, commodities and services trade, over the period 1990–2015.

Nevertheless, studies of this type are difficult to find, most of the research, in Europe, is focus on competitiveness of agricultural products [5,6], volatility of prices [7], sustainability of food system [8], the assessment of biological risk–diseases in animals [9,10], environmental risk [11], negative impacts of the agricultural commodities on ecosystems [12], and few analysis on commodities diversification and their impacts.

Regarding the analysis of the free trade agreements results in Colombia, there are several research about the economic and social impact of them, since the influence on the internal social conflict, reduction of the rural and urban poverty [13], analysis of the social dimension and labor rights in the frame of trade agreements [14,15]. Furthermore, protection of collective rights [16], considering the elements of safety, health, the environment, and free economic competition.

In the case of the Colombian agriculture sector, there is research on general descriptions of the impacts of the trade agreements in this sector as [17] analyzed the possible consequences to the country with the agreement with EU and United States. On the other hand [18], detailed the conditions of Colombian trade before and after the specific EU trade agreement, and mentioned the good performance of bananas and coffee exports in the first year of the agreement. There are reports of the fairtrade impacts in sectors as bananas [19], recent analysis of Colombian palm oil industry [20,21], as well, reports from institutions within the European Union and Colombia whose descriptions of the agricultural sector performance were generated.

However, specific research or analysis of agricultural commodity performance is not common. One of the reasons could be the lack of detailed statistics about the agricultural products trade because national databases do not have the reports for all agricultural categories and some of the export and import reports are shown as part of the global sector, without the description of the specific products traded with each partner. Even in international databases, such as UN Comtrade, some agricultural categories reported by Colombia do not have complete data, therefore, this research considered the trade reports of the EU.

Before developing the agricultural exports performance analysis, it is necessary to identify and understand general aspects of Colombian foreign trade and the current situation of the relationship with the European Union, the scope of the trade agreement, and the role of the agricultural sector in this bilateral trade relationship.

### Context of Colombian foreign trade

Historically, Colombian foreign trade has been based on the exploitation of natural and mining resources, ‘traditional exports’; among which hydrocarbons, coal, and ferronickel stand out, as well as agricultural products: flowers, coffee, and bananas. According to [22], endowments of relatively abundant factors (labor and land) have been extremely decisive, unqualified labor and natural resources, with low value added, are destined for export. By importing goods, they “correspond to resources scarce in the country, such as qualified labor, capital, and knowledge-intensive goods” [23].

However, the specialization on only these commodities or primary goods is not a correct strategy because they fluctuate frequently in international markets, affecting GDP growth and business cycles. Benefits and incomes of commodities from natural resources "depend on periods when there are good international prices" [24].

Colombia was one of the Latin American countries that, after the debt crisis in the 1980s, had to implement the economic stabilization and structural adjustment reforms set in the Washington Consensus, based on neoliberal principles and promoted by the World Bank and International Monetary Fund (IMF). The purpose of these programs was to accelerate trade liberalization so those countries would increase exports, according to their comparative advantages, and thus obtain foreign exchange to repay the debt. With the opening, it “was expected to find new markets that would increase the share of non-traditional exports” [24]. Therefore, Colombia moved from import substitution and protectionist policy, implemented 1930, began integration with global markets by 1970, with the promotion of exports, to finalize the arrival of the economic aperture in 1995, and the admission to the World Trade Organization (WTO).

According to national data, since Colombia’s insertion into the multilateral trading system as an official member of the WTO, the country has gone from exporting 10.201 million dollars in 1995 to 37.626 million dollars in 2008, with an almost uninterrupted growth, to $US60,125 million in 2012.

In the case of imports, Colombia has both complementary and substitution relationships with local production, with an emphasis on intermediate and capital goods. [25] cited studies that determined the Colombian export industry had not advanced as much as imports, which grew and diversified suppliers; meaning that the number of countries that exported to Colombia increased.

[22] mentioned that the Colombian foreign trade policy process, from protectionist to free trade policies, can be summarized in four strategic moments: 1) the beginning of the economic opening and the entry as an official member of the WTO in 1995; 2) the drop in exports caused by the world economic crisis of 2009; 3) the recovery and peak of exports in 2012; 4) the dramatic drop in exports in 2016.

Despite the transition, and Colombian strategic policies as a productive transformation, technical progress, enhanced products with elevated added value, and improving business productivity, [26] identified that the execution of foreign policy has not fully worked because it still depends on the export of primary products and substantial slowness in the execution of the productive transformation policies is evident. On the other hand, [2] point out that the country has been subject to the behavior of the mining sector, the problems of a lack of diversification and deindustrialization have intensified.

One of the challenges by most developing countries is the export basket diversification, since exports have an important role in the economic growth of countries, and “the free market trade policies have not achieved the expected results in Colombia in terms of diversification, one possible cause for this is its economy’s delayed internationalization” [27].

Between 2005 and 2015, Colombia registered an outstanding economic performance as a consequence of the increase in foreign investment in the raw materials sector, free trade agreements, reduction of trade barriers but mainly the oil and mining boom. However, this situation has not been a real development, since productivity and investment in sectors other than oil and mining have been insufficient; [28] argued that this is due to the heavy tax burden on investment, labor hiring, lack of infrastructure and financing access.

Understanding that the country assumes in its policies and programs the need to sustain long-term economic growth as an element to achieve the development, with directly impacts productivity, reduction of poverty, inequality, and unemployment.

Growth is associated with the production of goods and services, and development consists of improving people’s living conditions, satisfying their needs, thus, growth is the means and development is the end. [29] emphasize that growth is a necessary but insufficient condition for development, therefore, both processes are required and must complement each other.

In recent decades, mining raw materials has occupied a preponderant place in the country’s foreign trade and development model. [30] described that Colombia’s foreign trade depends on mining since the country exports mainly hydrocarbons and coal; although the exports of agricultural goods such as flowers, coffee, and bananas play a leading role in international trade, these are not yet decisive for the total exports of the country. It has not been possible to diversify and effectively increase exports from sectors different to mining; [24] mentioned that the share of the industrial and agricultural sectors have been decreasing in total national exports.

**Fig 1:**
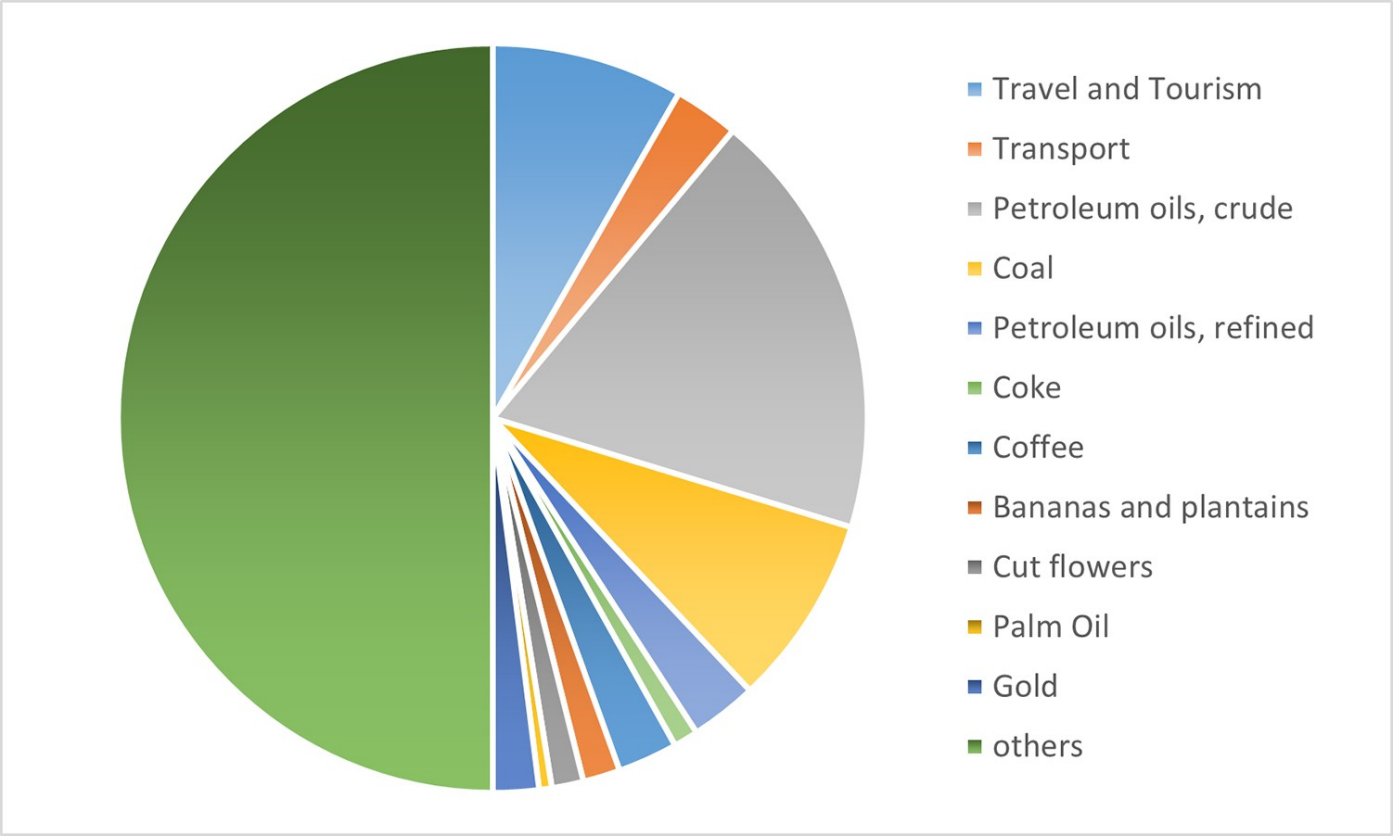
Exports of Colombia in 2018. Source: Prepared by the authors, based on data from The Atlas of Economic Complexity [31].

The following Fig 1. reflects the situation in 2018 concerning the sectoral concentration of global exports—around 44% of total exports are in the mining sector while agricultural commodities (coffee, bananas, cut flowers and palm oil) had a share of 12%, the category ‘other’ reflects the share of chemical, textiles, metals, machinery and electronic products.

[30] shows that Colombian foreign trade has been based, for years, on the exploitation of natural and mining resources because of factors mentioned previously and the country maintains a close commercial relationship with the same partners.

By 2019, data gleaned from the World Bank shows that the value of total national exports was $US51.12 billion, and, on the other hand, the total import value in the same year was $US70.9 billion, with a trade deficit of $US19.78 billion.; this negative trade balance has been constant since 2000. [30] signals that as a consequence, Colombia imports mainly industrial products, most part them do not produce locally, which prevents it from generating sufficient surpluses to invest and develop the productive apparatus in the country. Furthermore, imports have increased markedly, especially those from China.

Regarding the agricultural sector, according to data of the National Tax and Customs Directorate (DIAN) [32], in 2016, 11.155 companies exported merchandise to international markets for a value of $US31.095 million. Exports of the main mining companies on the list contributed 46% of total exports, more than $US14.552 million. On the other hand, [22] claims that the main non-mining exporting company in the country is the National Federation of Coffee Growers of Colombia, which contributes 2.1% of the total, $US663.2 million, the Unión Bananeros of Urabá stands out, with 1.3% of the total, just over $US397.2 million.

[33] mentioned that agricultural commodities should be highly competitive due to the availability of resources, nevertheless, the sector and rural areas present a big institutional challenge, and comprehensive policies are required that effectively diversify agricultural production, and exports within a stable and participatory institutional framework.

Transnational economies, the current weak institutional framework, and liberated international trade activities have exposed the fragile agricultural sector to a process of interrelation with external markets under conditions of productive and competitive inequality.

Concerning Colombian agriculture trade, information obtained from [31] The Atlas of Economic Complexity, let us appreciate the balance between export and import in terms of the amount of money and variety of products. By 2018, the export of agricultural products was $US7.78 billion, imports of $US7.61 billion; demonstrating that there is a close balance of trade in this sector, and not a large gap between exports and imports.

Related to the trade of products, the same database showed that the main agricultural commodities that Colombia exports were the categories of coffee, tea, spices, and bananas—plantains, which gathered 47% of total agricultural export. In the plant category (14%), there are cut flowers, followed by palm oil, sugar cane, and sucrose.

The share of some of these agricultural raw materials in the export world reflect contrasts because their participation in the country’s total exports is relatively low, however, those products occupy prominent places at the world level. Colombia is among the main worldwide exporters of agricultural commodities.

[22] higlighted that by 2018 Colombia has five main products with an important position in the global export trade of agricultural commodities, located among the top 10 in world exports. In the case of the trade in flowers, Colombia holds second place in global position with a share of 17%, and value of exports around $US 1.300.000, the third position with coffee (8.1% global share, $US 2.526.532), the fourth with bananas (8.4% and $US 914.937.000), the sixth position with palm oil (2.8% $US 208.586.000) and sugar cane (2.3%).

On the other hand, is important to recognize where the final export destinations and the provenance of the agricultural products are that arrive in the country. According to the Atlas of Economic Complexity database [31], the United States led both, as the first destination of Colombian agriculture products and the bigest seller of products of the same sector. The export trade is followed by the European Union, with around 13 importer countries which amount to approximately 28.94% of Colombian agriculture exports, less in proportion to South America and Asia.

In the case of Colombian agricultural commodity imports, these are led by corn with 12%, solid soybean residues, wheat, food preparation place third with 5%. Data from [31] shows that in 2018, the European Union had a participation of around 9% (Spain, Germany, the Netherlands, United Kingdom, Italy, France, and Belgium).

**Fig 2:**
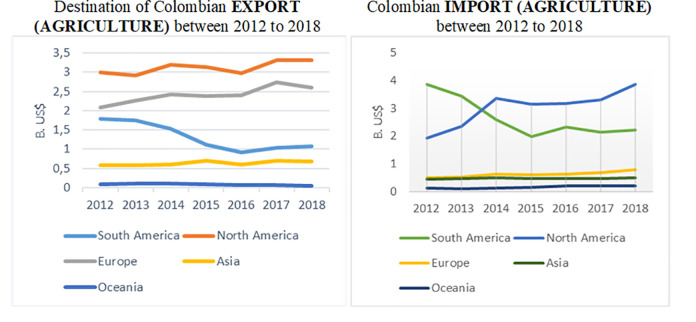
Colombian agricultural trade 2012–2018. Source: Prepared by the author, based on data from The Atlas of Economic Complexity [31].

The following Fig 2. reflects the huge participation of North America over recent years for Colombian agricultural imports. It also demonstrates that South America has been reducing its participation in that period, and Europe has a very low role in Colombian agricultural imports, beside its share in agricultural exports.

### Colombia–European Union trade relationship

The sectoral concentration of exports has been a continuous concern for governments in developing countries, according to [34]. Therefore, they promoted the creation of trade agreements to diversify markets, trade partners and increase exports. For its part, Colombia has adopted the strategy of implementing trade agreements; currently, it has 12 in force, among which the European Union (EU) stands out.

The most important element that defines this relationship between Colombia and the EU is the free trade agreement, which was signed on June 26, 2012, and has been in provisional application since August 1, 2013 and effective since November 5, 2014. According to the European Parliamentary Research Service (EPRS) [35], this agreement is denominated a fourth-generation agreement, since it not only facilitates access to the goods market by eliminating tariffs on EU and Colombian products, but it also provides access to services markets and investment, allows participation in the public procurement processes of both parties, and offers protection of intellectual property rights.

The new trade structure and the EU’s investment relationship approach promote broad social and environmental protection in Andean countries. [35] reports that this agreement involves commitments with social and environmental impacts, human rights, and sustainable development of partner countries. The aspects defined in the chapters of the agreement in which the EU requested from its partners (Colombia and Peru) involved the improvement of some fields for the continuity of the special trade relationship are thus: good labor conditions, guaranteeing human rights, the conservation of strategic ecosystems and the implementation of sustainable production processes, according to the F Elanbiz project [36].

The same project argues that the opening of the EU market to exporters from Colombia (liberalization in products and tariff concessions in agriculture) improves market access, promotes competitiveness, and innovation, and facilitates the trade and transfer of technology. The agreement entered into force seven years ago, being enough time for an assessment of operations within and trends for the agriculture sector, as well as commodities exported by Colombia to the EU.

**Fig 3:**
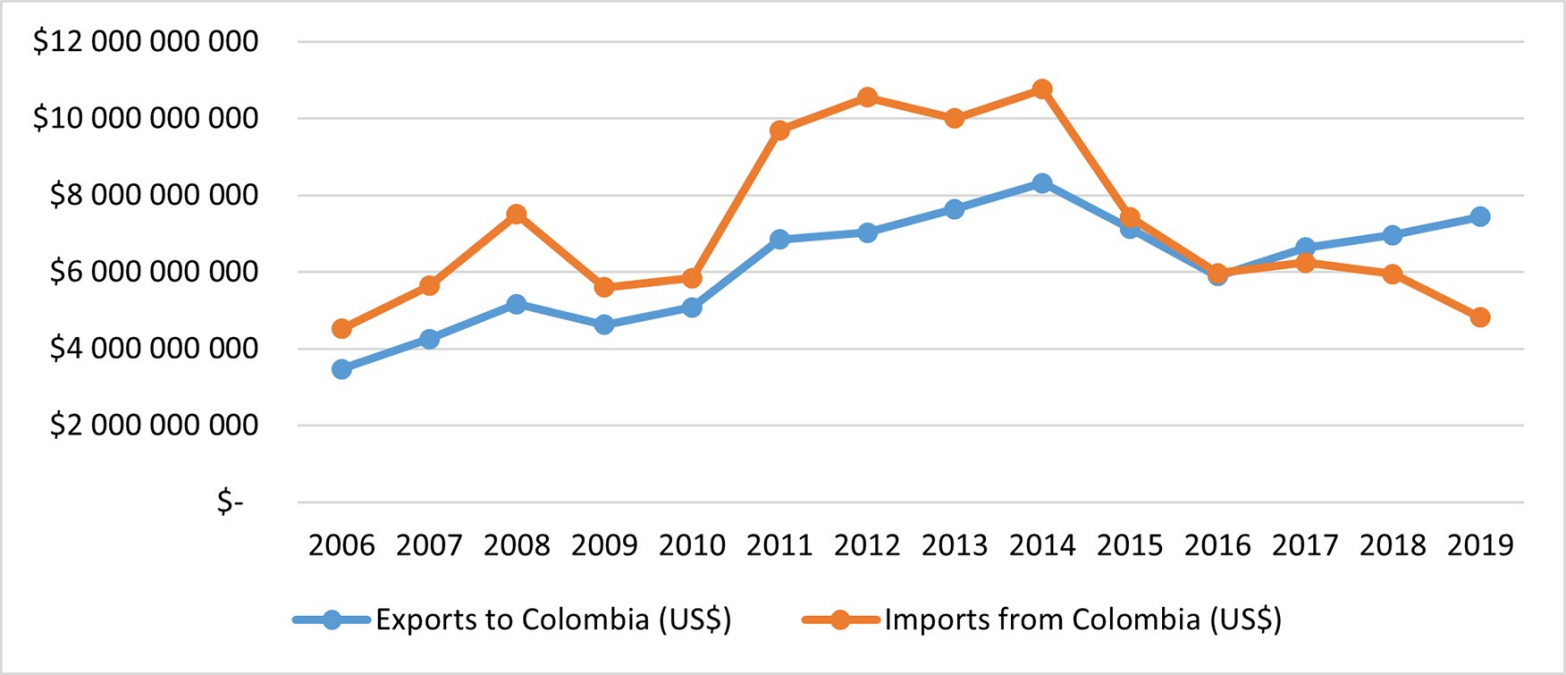
EU exports and imports with Colombia 2006–2019. Source: Prepared by the author, based on data from UN Comtrade database [37].

With the implementation of the agreement, an unexpected scenario occurred for Colombia—since the bilateral trade between Colombia and the EU, imports from Colombia have dropped 54% since 2012, as set forth in Fig 3. Before the agreement, the EU trade balance with Colombia had been in deficit, exports displayed growing trends but imports from Colombia had a higher trade value, and in 2012 the deficit achieved $US3.526 million. After that year, the trend became a surplus for the EU, maintaining the growth of exports to Colombia and decreasing imports; by 2019, the EU had a surplus of $US2.628 million.

Expectations with a trade agreement can be synthesized in four aspects: increase of exports, diversification of commodities exported, diversification of markets and partners—these are the main objectives and benefits that both parties are looking forward to achieving.

Six years after the implementation of the agreement, the Ministry of Trade of Colombia [3] recognized that the European Union is positioned as the third supplier of goods for Colombia, behind the United States and China. The EU represents 15% of the total imported, its export value maintains an increasing and continuous trend, as is appreciated in the previous Fig 3. However, Colombian results are not positive; its export value to the EU has been decreasing since 2014.

By 2018, Colombian exports were still concentrated in raw materials, with five basic products: coal (37.4%), bananas (13.6%), coffee (12.4%), petroleum (7.5%), and palm oil (7%), according to data from the Ministry of Trade. Therefore, there is no diversification of products, nor strengthening of sectors with value added, as [26] identified.

In the case of the EU exports to Colombia, the same report of the Ministry reported that the commodities traded in 2018 were electric machines (20.7%), medicines and immunological products (15.2%), airplanes and space vehicles (9.8%), tourism vehicles, and passenger transport (5.5%), and gasoline (1.4%), therefore, is appreciated that The EU exports involve industrial sector and products with value added.

[35] EPRS mentioned the significant drop in EU purchases coming from Colombia was in line with the drop in Colombian exports to the rest of the world. In the broader picture, there was a slowing down at that period in the economic growth of Latin American countries.

It is necessary to mention as well that new products were traded during the period the agreement entered into force (2013); 349 new products were exported to Colombia, mainly vegetable products, textiles, textile articles, and products of the chemical or allied industries sector.

Colombia exported 1,086 new products to the EU; these new products largely came from four non-traditional sectors: products of the chemical or allied industries (23.9% of new products), base metals and articles of base metal (16.4%), machinery, appliances, and their parts and accessories (15.4%), and textiles and textile articles (9.9%). Currently, they do not have an important share in the total export but began to participate in bilateral trade. It is highlighted that no new agricultural commodities with value added have been reported in the trade relation during this period.

### Cooperation and investment of European Union in Colombia

[36] claims that the European Union is the region that most foreign investment resides in Colombia; $US4.146 million in 2018—Spain was the country with the highest share (35%), followed by the United Kingdom (33%), Luxemburg (11%), and France (6%). Considering the strong investment from the EU in Colombia, the free trade agreement established conditions and strategic sectors for this Foreign Direct Investment.

The EU Trust Fund for Colombia is a strategy promoted in the agreement which has close to €127 million at its disposal from the EU budget and contributions of 19 EU Member States (Hungary, Croatia, Czech Republic, Cyprus, France, Germany, Italy, Ireland, Latvia, Lithuania, Luxembourg, Malta, the Netherlands, Portugal, Spain, Sweden, the United Kingdom, Slovakia, and Slovenia). Managed by the European Commission, and only EU Member donors, the Colombian Government, and the European Commission are allowed to propose actions with this fund. The European Union (2020) highlighted the fund and investments have among their aims to enhance access of EU companies to the Colombian market, encourage the consolidation of new economic sectors and demands, recognizing as a benefit for both parties the contribution to development of the region and creating new business opportunities and markets for EU companies in Colombia.

The Trust Fund mainly focuses on investment in rural development at a local level with projects aimed at improving land ownership, providing organizational support to producers, articulating public-private initiatives in rural areas, encouraging sustainable development—including the substitution of illicit crops—female entrepreneurship, food safety and access to basic services, and support with specialized technical assistance in areas that are required.

From this fund, Hungary and Colombia are developing cooperation with the ‘Sustainable Alternatives for Putumayo’ project; executed by the National Agricultural Research and Innovation Center (NAIK) and the Colombian institution Corpoamazonia, with the aim of ‘implementing sustainable models and eco-friendly alternatives to support the social and economic re-integration of local communities and post-conflict actors in the region of Putumayo, Colombia’ [38]. Agriculture sustainability training is offered along with assistance in environmental management, tourism, and the installation of drinking water infrastructure.

Regarding post-conflict investment, the European Commission reported [39] that the European Union has been supporting the sustainable implementation of the peace agreement by promoting the increase of production standards with satisfactory working conditions, protection of Colombian biodiversity, and environmental protections to create new markets in Colombia for EU products and services.

[36] presents the potential sectors the European Union has identified as needing improvement regarding the Colombian peace agreement are that of agro-industry, tourism (related to the environment), and infrastructure. According to analyses of the trade relationship of the EU and Colombia, some aspects of how these partners can achieve mutual benefits include food processing, tourism and the environment, and infrastructure.

### Agricultural commodities trade between Colombia and the European Union

In the trade balance of agricultural commodities between the EU and Colombia, there is a similar trend in European countries that stand out in export and imports trade. By 2018, according to [31], 75.8% of agricultural imports from Colombia had as main destinations six countries: Germany, the Netherlands, Belgium, United Kingdom, Spain, and Italy.

As was mentioned before, the most representative agricultural products that Colombia trade in the world are bananas, plantains, coffee, palm oil, and cut flowers, and the European Union is no exception—the majority of traded products from Colombia to the EU are agricultural raw materials: bananas, coffee, and palm oil.

Statistics reflect the increase of palm oil exports from Colombia to the European Union; to countries like the Netherlands and Spain, whom the most important agricultural commodity they receive from Colombia being palm oil, with 42% and 28,8% respectively of total agricultural imports [31]. However, in other countries such as Germany, Belgium, and Italy, bananas and coffee still led agricultural trade.

Regarding Colombian agricultural imports from Europe, this database shows the majority of trade products from the EU to Colombia are the commodity categories of food preparation, paper and paperboard, olive oil, and cellulose wadding. The countries that led this trade, by 2018, were Spain (23,35%), Germany (14,71%), the Netherlands (10,05%), and Italy (8,89%).

In the particular case of Hungary, the data reflects that its agricultural trade with Colombia is low, with a share of less than 1% in both export and import. In 2018, the gross export from Colombia to Hungary was $US605,000 that included: confectionery sugar (45.75%) and cut-flowers (45.34%). In the same year, Colombian imports from Hungary reached $US257,000 in the five main commodity categories of wood (29.47%), frozen vegetables (20.15%), other vegetables prepared or preserved (16.75%) and food preparations (12.31%).

## Methodology

The analysis of this paper focuses on the three main agricultural commodities exported from Colombia to the European Union which are bananas, coffee, and palm oil, and assesses the hypothesis posed: ‘rise of palm oil export trade from Colombia to the European Union has reduced the participation and value trade of Colombian traditional agricultural commodities “(bananas and coffee)”. To develop this, four components were analyzed—their trade value, exports share, the volume of production, and the correlation coefficient with the total Colombian agricultural export to the EU in the period of 2008 to 2019.

Collection of the agricultural commodities data was realized in international databases, considering the standard categories from the FAO international product classification for agricultural statistics, which bases the list on Central Product Classification (CPC) of the United Nations and Harmonized System (HS)—Harmonized Commodity Coding and Classification System.

From FAO’s classification [40], the following groups were prioritized that gather together coffee, bananas, and palm oil products.

- *Banana and plantain* (Group: Fruits and derived products)
- Bananas, including plantain fresh or dried
- *Palm oil* (Group: Oil-bearing crops and derived products)
- Palm oil and its fractions, whether or not refined but not chemically modified
- *Coffee* (Group: Stimulant crops and derived products)
- Coffee, tea, mate, and spices

The indicators of trade value and production (export quantity) of these three categories were found in European Union database reports, and in the case of Colombia, there was no specific report, only global data was available of agricultural commodity exports and imports. Therefore, the data used in this analysis comes from the UN Comtrade database, with the EU as a reporter, and The Atlas of Economic Complexity-Growth Lab, Harvard University.

Data was collected from 2008 to 2019 in order to identify the performance and share of those agricultural commodities exported from Colombia to the EU; a period of time that covers before and after the free trade agreement, considering not exclusively the economic value of export but the quantity of products traded as well.

The last component of this analysis is the correlation coefficient, an important statistic tool to identify association, linear relationship and the strength between observed variables, (dependent variables), in this case, the total agricultural export value, and independent variables such as banana, coffee and palm oil exports. The correlation coefficient “provides information about not only the strength but also the direction of a relationship” [41]. For that reason, was considered this analysis of agriculture commodities.

Correlation coefficients are scaled in a range from –1 to +1, where 0 indicates that there is no linear or monotonic association. “The strength of the correlation is not dependent on the direction of the relationship” [42] if it is positive or negative, since a coefficient value of < 0.1 indicates a negligible relationship and >0.9 indicates a very strong relationship.

The significance (p) of the correlation, according to [42], indicates that if the observed sample data provides ample evidence to reject the mull hypothesis, an association between variables can be considered statistically significant if P> 0.05.

To build the correlation coefficient of banana, coffee and palm oil exports in the total agriculture commodity exports from Colombia to EU, statistical software was used to organize and analyze data: IBM SPSS, using the bivariate Pearson Correlation.

## Results and discussion

**Fig 4:**
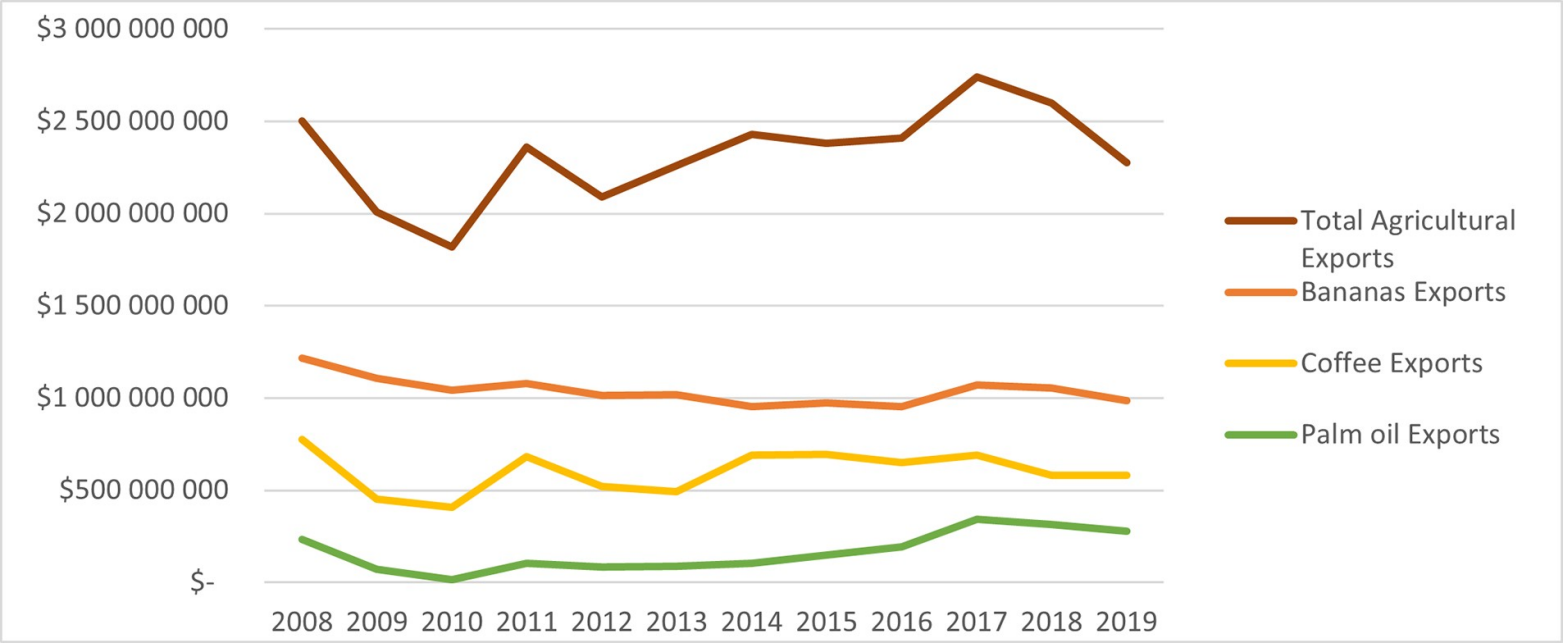
Trade value of Colombian agricultural commodities exported to the European Union. Source: Prepared by the author, based on data from UN Comtrade [37] and Atlas of Economic Complexity [31].

The first segment of the analysis of agricultural commodities considers the trade value indicator of the bananas, coffee and palm oil categories in the exports from Colombia to the EU in the period from 2008 to 2019. Fig 4 reflects the performance of each commodity, compared to the total Colombian agricultural export value.

It was observed that bananas have been leading agricultural exports to the EU for a long time, however, without a continuous and strong growth. There was a decreasing trend until 2016, when national exports had a short period of incline.

Against the perception that the main agricultural export product of Colombia is coffee, it is appreciated that bananas have been the most important export agricultural commodity with the EU over the recent period, with a large difference in production and export value over other commodities. Considering that the European Union is the main buyer of Colombian bananas, approximately 82% of the total exports of this product reach Europe, according to data of Ministry of Trade of Colombia [3].

By 2019, around 43% of Colombian agricultural exports to Europe were bananas with just 25% being coffee. It is possible to identify that coffee exports have not increased over the 10 years analyzed. Its export performance was volatile, with periods of ups and downs, without a significant increase of export value since the implementation of the trade agreement in 2013 ($US491,237,468) to 2018 ($US579,133,300).

The coffee market, as earlier mentioned, displayed volatile behavior over time as [43] mentioned, due to the variables that define its economy, such as the trends of international prices. Despite 80% of Colombian coffee produced having as final destination export market, the United States is the main destination of most of the export production. [44] claimed that an important factor is a strong relationship between this country with some of the biggest Colombian coffee exporters and the Coffee National Federation.

In Europe, for its part, by 2017/18, Colombia was in third position as a coffee trader with the EU, behind Brazil (31%), Vietnam (24%), then Colombia (7%), and Honduras (6%), according to data of USDA [45]. This situation and the trend of export values reflect that despite the agreement, there is a lack of efficient strategies to promote the exports of competitive Colombian coffee and its diversified commodities to the EU.

There are four important years to consider in the analysis of the commodities export value trend—2009 with the drop in exports as a consequence of the world economic crisis, 2013 when the free trade agreement was incorporated, 2016 with the drop of Colombian exports because of the contraction of the national economy and with the reduction of oil prices, following by a contraction in 2019, resulting in low trade values of export products.

Between 2012 and 2018, the total exports of Colombian agricultural commodities to the EU achieved an increase of 25%, mainly motivated by “the growth of fruit sales, incorporation of new fruits including Gulupa, and Lima Tahiti” [3]. In 2012, the Hass Avocado was not on the export list from Colombia to the EU, and with the free trade agreement, it began to be traded. By 2018, the export value was $US60.6 million.

On the other hand, the situation of Palm oil exports from Colombia to the EU shows an increasing trend since 2010, from an export trade value of $US16,458,489 in 2010 to $US313,247,425 in 2018. By 2019, there had been a reduction in the trade value ($US278,326,701). However, the export quantity traded was higher than the year prior; 463.446.645 Kg in 2018 to 490.366.017 Kg in 2019, and the same situation happened to bananas that in 2019 (1.471.719.474 Kg), more quantity was traded than in 2018 (1.452.265.283 Kg), but the trade value of its export decreased. Therefore, it is important to highlight that the export quantity of palm oil to Europe in 2019 maintained an increasing trend but the trade value was reduced, affected by international factors.

Compared with the other two traditional commodities (coffee and bananas), palm oil presents the biggest and most continuous growth of export trade values since the implementation of the free trade agreement with the EU in 2013. By 2018, palm oil exports were 252% higher than in 2012.

**Fig 5:**
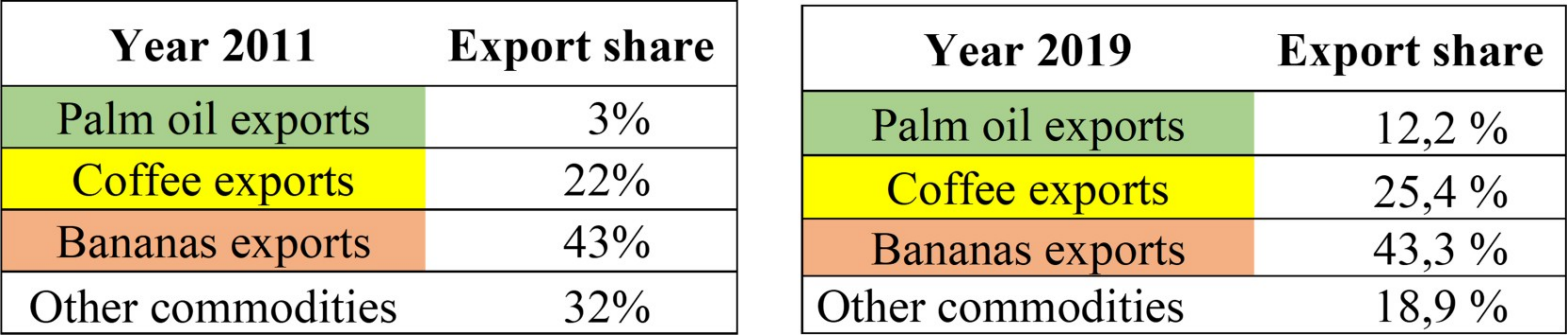
Export value share of Colombian agricultural commodities with the EU. Source: Prepared by the authors, based on data from UN Comtrade [37] and Atlas of economics complexity [31].

This situation is reflected in the export value share of Colombian agricultural commodities as well, with a parallel that was posed between the share of products exported to the EU in 2011 and 2019 (Fig 5).

The increase of Palm oil exports in bilateral trade can be identified in the above tables. Banana exports kept almost the same share eight years later, and in the case of coffee exports, growth was small—from 22% in 2011 to 25.4% in 2019. The other commodities, related in the export share figure, gather together the categories of fresh fruit, avocados, sugarcane products, cut flowers, coconut and palm kernel oil, among others, and their export share was reduced by 2019 as well, reflecting a decreasing in the diversification of agriculture commodities traded from Colombia to the EU.

**Fig 6:**
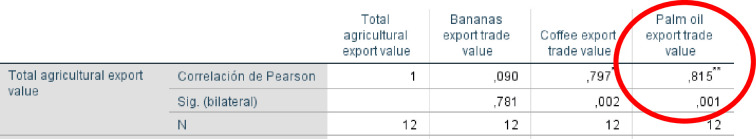
Correlation of agriculture commodities trade value. Source: Own calculations.

The last component to evaluate palm oil performance in the export trade with the EU for the period (2008–2019) was developed with the correlational coefficient in the SPSS program to identify the significance, the strongest and weak relationship between the commodities (coffee, bananas, and palm oil) and the total Colombian agricultural export trade. The level of linear association between the two variables (dependent and independent) was measured by utilizing the Pearson Correlation (Fig 6).

The result of the correlation in commodities trade value reveals that palm oil obtained the highest value of Pearson Correlation coefficient (0.815), which means a strong and positive relationship, thus, the export value of palm oil had a strong and positive impact on the growth of total agricultural export values to the EU. In that period, palm oil contributed to the increase in agricultural trade values from Colombia to the EU in contrast to the correlation of the coffee and bananas with the lowest Pearson Correlation values, reflecting a compact relationship, mainly bananas, with an increase of Colombian agricultural export values to the EU.

Regarding the (p) or significance of the correlation, palm oil has the lowest value of 0.001. Therefore, palm oil has the most significant linear relationship with Colombian agricultural export values. This means that the performance of palm oil export trade value had the highest and most positive influence on increasing the total of Colombian agricultural exports to the EU between 2008 and 2019, in comparison with the performance of coffee and bananas.

**Fig 7:**
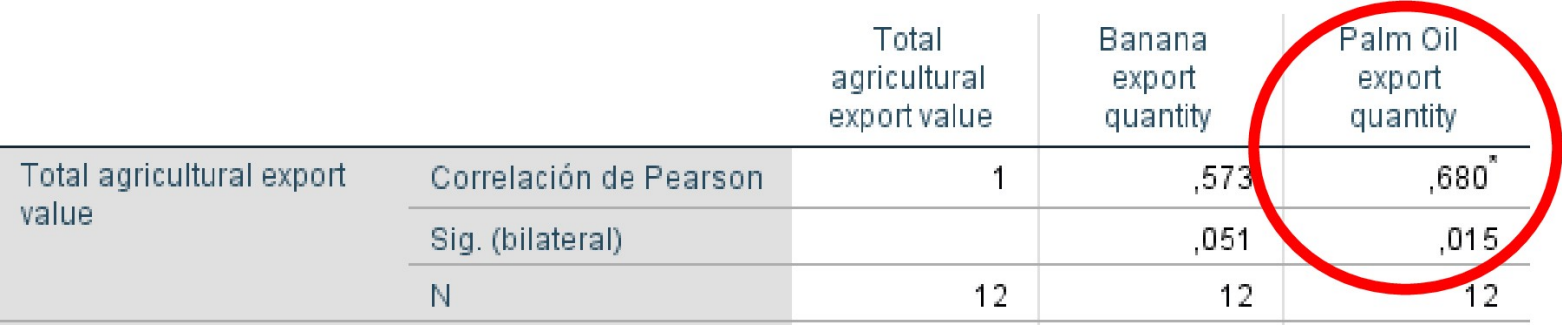
Correlation of agriculture commodities production. Source: Own calculations.

Considering the importance of analyzing trade values as the quantity of exports traded, the correlation for the indicator of production (export quantity) was developed. The correlation was executed with bananas and palm oil commodities, since data of the Colombian Coffee export quantity to the European Union was not present, therefore, this analysis was possible only with two of the commodities (Fig 7).

Yield results favorable to palm oil, with the highest Pearson Correlation value (0,680) and the significance (0.015) being lower than 0.05. This is interpreted as exhibiting a strong positive and significant relationship between palm oil export production quantity and the Colombian agricultural export value to the European Union between 2008 and 2019.

## Conclusions

The three indicators of export quantity, trade value, export share, and the statistical analysis of correlation coefficient provided important data to identify the performance of the three most traded Colombian agricultural products to the European Union (bananas, coffee and palm oil). Moreover, the indicators let determined the impact and influence of each of the products in the total exports of the Colombian agricultural sector, from 2008 to 2019, considering the implementation of the free trade agreement.

- With the rise of palm oil exports to the EU, coffee and bananas retained participation in exports to the EU, coffee has a slight increase in its share, and for its part, banana export value kept the same share at the end of the period analyzed. Bananas as a commodity continue as the leader, with the EU as the destination of 80% of the total exports of Colombian bananas. Therefore, the hypothesis is not completely true, since an increase in palm oil exports does not reduce the share of the two traditional commodities, but terminates the growth in export value of them, and palm oil became the export commodity with the biggest and most uninterrupted growth since the implementation of the free trade agreement with the EU.
  The reduction of export participation was significant in the category ‘other commodities’, in which the change in export share was acknowledged; from 32% in 2011 to 18.9% in 2019, reducing the possibility of achieving agricultural export diversification and creating more dependence on the 3 main commodities (bananas, coffee and palm oil).
- Colombian coffee as a commodity represents important participation in agricultural trade with the European Union, has second position in export products, and is one of the most recognized Colombian traditional products in the world. Nevertheless, the trend of its export value to the EU is decreasing since 2014. Coffee exports are concentrated on partners like the United States and are reducing their participation in EU markets. By 2017/18, Colombia had the third position of coffee trader with the EU.
- It was confirmed with the correlation coefficient that the palm oil export value performance has a strong and positive impact on the growth of the Colombian total agricultural exports to the EU. Between 2008 and 2019, palm oil was the commodity that contributed the most to the increase of agricultural export value from Colombia to the EU, in contrast to the correlation of coffee and bananas that had the lowest Pearson Correlation values, reflecting a compact relationship with agricultural export values to the EU.
- Between 2012 and 2018, the total exports of Colombian agricultural commodities to the EU achieved an increase of 25%, motivated by the growth of fruit sales, incorporation of new fruits, for instance avocados, and the increase in palm oil exports in 2018 was 252% higher than in 2012. Moreover, the share of palm oil in the total agricultural export value of Colombia moved from 3% in 2011 to 12.24% in 2019. Compared with the two traditional commodities (coffee and bananas) palm oil presented the biggest growth of export value since the ratification and entry into force of the free trade agreement with the EU in 2013.
- Despite the economic aperture and implementation of free trade agreements, Colombia continues with its dependency on traditional commodities, and raw materials or basic products. In the agricultural trade relationship with the European Union, there is not a strong diversification of exports with value added, deindustrialization has been intensified because the manufacturing infrastructure has not been modernized. Those aspects are needed to be consider in the strategies and programs of the Colombian ministries of agriculture and foreign trade.
- Colombian agricultural raw materials have low participation in the country’s total exports; however, those commodities are recognized and occupy prominent places at the world level. Colombia is among the top 10 worldwide exporters of five agricultural commodities (flowers, coffee, bananas, palm oil, and sugar cane).
- In the analysis of export trade commodities, it is recommended to consider the indicators of value of trade, export share and the export quantity of the products since, in the analysis of palm oil and bananas, it was recognized that the reduction of export trade value does not always mean reduction of the production and the export quantity traded. A reduction in the value of palm oil and bananas from 2018 to 2019 was recognized, however, the amount of product traded in 2019 was bigger than in the previous year Therefore, the value paid for the exports was less but the production was bigger. It is necessary to include these three factors in the analysis of export commodities to better understand the scenarios.
- The results of the correlation analysis done in this paper, and the identified trends of production and trade value for the commodities is useful data for decision makers of agricultural and foreign trade institutions in Colombia and the European Union, to design strategies, programs and projects of investment.
